# Influence of Diet, Menstruation and Genetic Factors on Iron Status: A Cross-Sectional Study in Spanish Women of Childbearing Age

**DOI:** 10.3390/ijms15034077

**Published:** 2014-03-06

**Authors:** Ruth Blanco-Rojo, Laura Toxqui, Ana M. López-Parra, Carlos Baeza-Richer, Ana M. Pérez-Granados, Eduardo Arroyo-Pardo, M. Pilar Vaquero

**Affiliations:** 1 Department of Metabolism and Nutrition, Institute of Food Science, Technology and Nutrition (ICTAN), Spanish National Research Council (CSIC), C/José Antonio Novais 10, Madrid 28040, Spain; E-Mails: ruth.blanco@ictan.csic.es (R.B.-R.); laura.toxqui@ictan.csic.es (L.T.); granados@ictan.csic.es (A.M.P.-G.); 2 Department of Toxicology and Health Legislation, Faculty of Medicine, Complutense University of Madrid, Plaza de Ramón y Cajal, Madrid 28040, Spain; E-Mails: amlopezparra@med.ucm.es (A.M.L.-P.); cbaezaricher@med.ucm.es (C.B.-R.); earroyopardo@med.ucm.es (E.A.-P.)

**Keywords:** iron deficiency, red meat consumption, menstrual blood loss, SNP, *HFE* gene, menstruating women

## Abstract

The aim of this study was to investigate the combined influence of diet, menstruation and genetic factors on iron status in Spanish menstruating women (*n* = 142). Dietary intake was assessed by a 72-h detailed dietary report and menstrual blood loss by a questionnaire, to determine a Menstrual Blood Loss Coefficient (MBLC). Five selected SNPs were genotyped: rs3811647, rs1799852 (*Tf* gene); rs1375515 (*CACNA2D3* gene); and rs1800562 and rs1799945 (*HFE* gene, mutations C282Y and H63D, respectively). Iron biomarkers were determined and cluster analysis was performed. Differences among clusters in dietary intake, menstrual blood loss parameters and genotype frequencies distribution were studied. A categorical regression was performed to identify factors associated with cluster belonging. Three clusters were identified: women with poor iron status close to developing iron deficiency anemia (Cluster 1, *n* = 26); women with mild iron deficiency (Cluster 2, *n* = 59) and women with normal iron status (Cluster 3, *n* = 57). Three independent factors, red meat consumption, MBLC and mutation C282Y, were included in the model that better explained cluster belonging (*R*^2^ = 0.142, *p* < 0.001). In conclusion, the combination of high red meat consumption, low menstrual blood loss and the *HFE* C282Y mutation may protect from iron deficiency in women of childbearing age. These findings could be useful to implement adequate strategies to prevent iron deficiency anemia.

## 1. Introduction

Iron deficiency anemia remains a major worldwide health problem in the 21st century. This fundamental health issue has still not been resolved and continues affecting the health, quality of life and working capacity of approximately two billion people all over the world, especially children and women of child-bearing age [1,2]. The principal associated consequences, fatigue and reduction of work performance in adults, could have substantial health and economic costs [3]. Therefore, it is important to study the aetiology of iron deficiency anemia, which can be caused by a wide variety of risk factors.

It is well established that low iron intake and low bioavailability of dietary iron are crucial in the development of iron deficiency [4]. The bioavailability of non-haem iron, the main source of iron from most diets, is affected by the presence of several dietary components. Non-haem iron absorption is enhanced principally by animal tissue and ascorbic acid; whereas phytic acid and polyphenols are the main inhibitors. On the contrary, haem iron (10%–15% of total iron intake) is absorbed more efficiently [5].

Another important factor that contributes to a negative iron balance in women at fertile age is menstruation. The most accurate method for measuring menstrual blood loss (alkaline haematin method) is the chemical analysis of the blood content of used sanitary products [6] but is tedious, not appropriate for widespread diagnostic use as women have to collect and store their entire sanitary products, and in addition it has been suggested that up to 12% of menstrual blood loss could be loss extraneously and non-quantified [7]. Other methods are based on questionnaires to estimate menstrual blood losses [8]. However, few reports show the relationship between menstrual blood losses and iron status [9–11].

Finally, it has been suggested that genetic factors also play a role in iron status, and recent genome wide association studies have found associations between the presence of genetic variants and iron status biomarkers [12–14]. Our research group observed in Spanish menstruating women that a large percentage of the genetic variation of serum transferrin was explained by two Single Nucleotide Polymorphisms (SNPs) located in the transferrin (*Tf*) gene and two in the haemochromatosis (*HFE*) gene [15]; and that the SNP rs1375515, located in a calcium channel gene (*CACNA2D3*), may play important roles in iron status [16].

The influence of all these factors on iron status has been studied in menstruating women, but in a separate manner. Therefore, the aim of this study was to investigate the combined influence of diet, menstruation and genetic factors on iron status in healthy Spanish women of childbearing age.

## 2. Results and Discussion

To our knowledge, this is the first study that investigates, by a cluster approach method, the aetiology of iron deficiency by evaluating simultaneously the impact of diet, menstrual blood loss and genetic factors on iron status of menstruating young women.

There are several laboratory tests that can be used to assess iron status, nevertheless, the absence of a definitive and unique biomarker of iron status make it difficult to associate risk factors with different degrees of iron deficiency [2,17]. Using *k*-means cluster analysis of 6 iron status parameters (haemoglobin, ferritin, soluble transferrin receptor (sTfR), transferrin, serum iron and red blood cell distribution width) three distinct groups were identified (Table 1). The age of the participating women was 25 ± 5 years (mean ± SD) and body mass index 21.7 ± 2.5 kg/m^2^.

Differences between the clusters were highly significant for all parameters by ANOVA (*p* ≤ 0.001), and significant differences were seen between Cluster 1 (*n* = 26), Cluster 2 (*n* = 59) and Cluster 3 (*n* = 57) by Bonferroni *post hoc* test (*p* ≤ 0.01), except for transferrin and serum iron. Higher levels of transferrin were shown for Clusters 1 and 2 with respect to Cluster 3 (*p* < 0.001), without differences between them. Concentrations of serum iron were similar in Clusters 2 and 3, but significantly higher than in Cluster 1 (*p* < 0.001).

Therefore, we identified three iron metabolic phenotypes: women with poor iron status close to developing iron deficiency anemia (Cluster 1); women with mild iron deficiency (Cluster 2) and women with normal iron status (Cluster 3).

Regarding dietary factors, there were no differences between clusters for the intake of energy and nutrients: proteins, carbohydrates, lipids and total iron (Table 2). Moreover, there were no significant differences in the consumption of several food groups: cereals, legumes, vegetables, fruits, milk and dairy products, fish, eggs, white meat, and processed meat; except for red meat consumption (*p* < 0.01). Significantly higher red meat intake was observed in Cluster 3 compared to Cluster 2 (*p* < 0.05, Bonferroni *post hoc* test).

It is remarkable that total iron intake of the 3 clusters was similar although iron status varied markedly. This is in agreement with previous findings [18–20], and highlights the importance of consuming haem iron, highly bioavailable, to improve iron status [21]. This is supported by the significant differences observed in red meat consumption between clusters, since it has been estimated that about 40% of iron content in this food is haem iron [22]. On the contrary, white and processed meat intakes seem not to enhance iron status, as there were no differences among clusters. These results make sense in terms of white meat consumption, as this type of meat contains lower amounts of haem iron than red meat. The fact that processed meat had no influence on iron status is an unexpected finding, which may be explained as processed meat products normally contain ingredients from cereals that can contribute greatly to total iron but mainly supply non-haem iron. These results have not been observed before, since other authors studied the influence of total meat intake, or red and processed meat compared to white meat consumption on iron status [23,24].

With respect to the menstrual blood loss assessment according to clusters (Table 3), there were no differences in the age of menarche, the menstrual cycle length, the duration of the menstruation, the days with intensive bleeding or the number of tampons and/or towels used in the day of more intensive bleeding (TTI). However, a significant difference was observed in the MBLC, which was lower in Clusters 2 and 3 with respect to Cluster 1. Also, it was observed that the percentage of oral contraceptives (OC) users was significantly higher in Cluster 3 with respect to Clusters 1 and 2. This highest percentage of women that use OC together with the lowest MBLC, confirms previous findings of an inverse relationship between OC and menstrual blood losses [25].

Menstrual blood loss has been shown to be an important determinant of iron status in young women [26]. We designed a detailed questionnaire, easy to complete and analyze, to determine a MBLC that was highly related to the iron status of the volunteers. Our results are in agreement with one study that measured menstrual blood loss in a quantitative way [27]. They also agree with Heath *et al*. [11] who worked with a similar coefficient that was correlated to serum ferritin levels, but did not observe differences between women with mild iron deficiency and normal iron status.

Concerning the genetic factors, genotype frequencies of rs3811647, rs1375515, rs1799945 (H63D), rs1800562 (C282Y) and rs1799852 were similar among the clusters and no differences were observed (Table 4).

We previously observed that the minor allele of rs3811647 located in the *Tf* gene was related to high serum transferrin and low transferrin saturation [15] and the minor allele of rs1375515, located in the calcium channel *CACNA2D3*, was associated with low levels of haemoglobin, mean corpuscular volume, and ferritin [16], suggesting that both SNPs play a role in iron deficiency anemia risk. The other three SNPs, rs1799852 in the *Tf* gene and the H63D and C282Y substitutions exhibited a counterbalancing influence [15,16]. The difference between previous results and those of the present study should be interpreted considering that the volunteers were healthy and anemic women were excluded. In this regard, it is remarkable that no women in Cluster 1 were heterozygous for the C282Y mutation nor mutated homozygous for the H63D position. This is in agreement with the suggested protective effect against iron deficiency anemia of the *HFE* gene mutations [15,16].

Results of the categorical regression test are shown in Table 5. Three independent factors were included in the model that better explained cluster belonging. Model characteristics were: *n* = 142, *R*^2^ = 0.142 and *p* < 0.001. The most influential factor was red meat consumption (*p* = 0.001 and importance coefficient = 0.677); higher red meat intake predicts better iron status. Menstrual blood loss coefficient was inversely related to clusters belonging, therefore, the higher menstrual blood loss coefficient, the lower iron status (*p* = 0.085 and importance coefficient = 0.170). Thirdly, presence of the C282Y mutation was related to higher iron status (*p* = 0.095 and importance coefficient = 0.153).

The present study displays a model which shows that red meat intake, menstrual blood loss and a genetic variant from the haemachromatosis gene (C282Y), all play an important role in the iron status of Spanish women of childbearing age. Several authors have studied the influence of some of these factors on inter-individual variation in iron stores. Heath *et al*. [11] and Harvey *et al*. [27] observed that menstrual blood loss and the type of diet were significant predictors of iron status in a group of young women from New Zealand and UK respectively, but they did not analyze genetic variants. On the contrary, Cade *et al*. [28] determined that the *HFE* mutation C282Y together with haem iron intake and postmenopausal status were associated with higher ferritin levels in a group of middle age women.

This investigation has public health implications, as it reveals the most important factors involved in the aetiology of iron deficiency anemia in a vulnerable population group. All dietary, gynaecologic and genetic factors should be considered in a combined manner in order to take the most effective measures to improve iron status and prevent the development of iron deficiency anemia. It was obtained that the factor that had the highest influence on iron status is red meat consumption. This result is very favorable for prevention strategies planning, since diet is a modifiable factor, in contrast to genetics and menstrual blood loss (genetically determined) [29]. Therefore, frequent consumption of red meat could be promoted to improve the iron status of healthy women of childbearing age, in the context of a healthy diet.

Consequently, we consider that this cluster approach could be useful to detect different degrees of iron status among the population groups, especially those at risk of iron deficiency anemia. Further studies should be carried out including anemic women.

## 3. Experimental Section

### 3.1. Subjects

A group of 142 healthy menstruating women was selected. The volunteers, aged 18–35 years, had to be Caucasian, non-smokers, non-pregnant and non-breastfeeding. Subjects were excluded from the study if they had amenorrhea, menopause, iron deficiency anemia, thalassaemia, haemochromatosis, chronic gastric diseases (inflammatory bowel disease, Crohn disease, gastric ulcers, celiac disease, haemorrhagic diseases), or renal disease, blood donors and women who regularly consume iron supplements. The participants signed a written informed consent to a protocol approved by the Clinical Research Ethics Committee of Hospital Puerta de Hierro, Madrid, and the Spanish National Research Council Committee, Madrid, Spain.

### 3.2. Blood Sampling and Biochemical Assays

Blood samples were collected by venipuncture after a 12-h fasting period, between 08:00 h and 09:00 h. Serum and plasma were obtained after centrifugation at 1000× *g* for 15 min and stored at −80 °C. Haematocrit, mean corpuscular volume (MCV) hemoglobin and red blood cell distribution width were determined following standard laboratory techniques using the Symex NE 9100 automated haematology analyzer (Symex, Kobe, Japan). Serum iron, serum ferritin and serum transferrin were determined by the Modular Analytics Serum Work Area analyzer (Roche, Basel, Switzerland). Transferrin saturation (%) was calculated as follows: serum iron (μmol/L)/TIBC (μmol/L) × 100, where TIBC is total iron binding capacity, calculated as 25.1 × transferrin (g/L). Serum soluble transferrin receptor (sTfR) concentration was determined using an enzyme inmunoassay technique (sTfR Human ELISA; Biovendor, Heidelberg, Germany). Automated determinations were subjected to the ISO 9001-2000 requirement. The intraassay coefficient of variation (CV) of sTfR determination was 3.5% and the inter-assay CV was 4.3%.

### 3.3. Dietary Control and Anthropometric Measures

Each subject’s dietary intake was assessed by a 72-h detailed dietary intake report (3 consecutive days including one holiday), previously validated and proved valuable to assess nutrient and food intakes [30]. The volunteers were instructed to specify the types of food consumed, including alcoholic beverages, coffee and tea, and the serving weights. Daily food, energy intake, total iron intake and energy provided by macronutrients were calculated with a computer application using the Spanish Food Composition Database (DIAL, Alce Ingeniería; Madrid, Spain). Meat and meat products intake was divided into red meat (including beef, lamb, veal and pork), white meat (including chicken and turkey) and processed meat (including cured and smoked meats, ham, bacon, sausages, chorizo, salami, *etc*.). Total grams of red, white and processed meat were calculated taking into account both fresh cuts and meat consumed as part of a composite meal.

Anthropometric measures were taken using standardized procedures. Body weight was measured with a calibrated Seca scale (to a precision of 100 g) and height was measured with a stadiometer incorporated into the scale. Body mass index (BMI) was calculated as weight/height squared (kg/m^2^). Systolic and diastolic blood pressure was measured with a validated digital automated blood pressure monitor (OMROM M6, Omrom Health Care Co., Ltd., Kyoto, Japan).

### 3.4. Menstrual Blood Loss Assessment

Menstrual blood loss of each woman was assessed by a questionnaire designed by the research group. Volunteers were asked about their menarche age, oral contraceptives (OC) use and menstrual cycle regularity. At baseline and during 4 months, volunteers had to write down during the menstrual period: the starting date, the duration of the menstruation, the number of days with intensive bleeding, and the number of tampons and/or towels used in the day of more intensive bleeding (*TTI*). Participants were asked to carry the questionnaire with them, and not to fill it out from memory.

The menstruations per year (*M*y), the number of tampons and/or towels used in the days of more intensive bleeding per year (*TTI*y), and the number of tampons and/or towels used in the days of less intensive bleeding per year (*TTNI*y) were calculated as follows:

My=365/duration of the menstrual cycle (days)

where the duration of the menstrual cycle is the number of days between the start dates of two consecutive menstrual periods.

$$\begin{array}{c} TTI\text{y}=TTI\times \text{days with intensive bleeding}\times M\text{y}\\ TTNI\text{y}=TTI\times 0.15\times (\text{duration of the menstruation }(\text{days})-\text{days with intensive}\;\text{bleeding})\times M\text{y} \end{array}$$

This last calculation took into account that approximately 10%–20% of menstrual blood loss is produced after the days of more intensive bleeding [8].

Finally, a Menstrual Blood Loss Coefficient (*MBLC*) was determined as follows:

MBLC=TTIy+TTNIy

### 3.5. Genotyping

DNA was extracted from whole blood using standard phenol-chloroform methodology with proteinase K. Five SNPs known to be related to iron metabolism were selected [17,18]. Two of them (rs3811647, rs1799852) are located in the *Tf* gene, other two (rs1800562 or position C282Y and rs1799945 or position H63D) are located in the *HFE* gene and the last one (rs1375515) is found in the *CACNA2D3* gene. Genotyping of 5 SNPs was carried out by a minisequencing method previously reported [31].

### 3.6. Statistical Analysis

Data are presented as means with their standard deviations. Iron status biomarkers values were *Z* score-transformed and a cluster analysis was performed using the *k*-means algorithm. The final number of clusters was fixed as three, taking into account cluster proximities for each cluster center and to ensure minimum error in cluster membership. Red, white and processed meat intake values (g/day) were log-transformed for statistical testing. Analysis of variance (ANOVA) was used to compare iron biomarkers, food and energy and nutrients intake, and menstrual blood loss parameters according to the clusters. Where statistically different effects were found (*p* < 0.05) the Bonferroni *post hoc* multiple comparison test was used. Genotype data quality was verified for each SNP by testing for Hardy-Weinberg equilibrium. A crosstabs procedure and the Chi-squared test were used to estimate if there were differences between the genotype frequencies distribution and the OC use among clusters. A categorical regression with optimal scaling (CATREG) was performed to identify the dietary, genetic and physiological risk factors associated with cluster belonging. The non-significant and unimportant factors were stepwise eliminated from the regression models. Elimination criteria, in this order, were *p* > 0.1 and importance coefficient <0.05. Data were analyzed using the SPSS statistical package for Windows (version 19.0; SPSS Inc., Chicago, IL, USA).

## 4. Conclusions

In conclusion, we found, by a cluster approach method, that three factors: red meat consumption, menstrual blood loss and the C282Y mutation of the *HFE* gene, all play a significant role in the iron status of menstruating women. These findings could be useful to implement adequate strategies to prevent iron deficiency anemia in population at risk.

## Figures and Tables

**Table 1.** Iron biomarkers according to the clusters.

Parameter	Cluster 1	Cluster 2	Cluster 3
Haemoglobin (g/dL)	12.45 ± 0.79 ^a^	13.06 ± 0.76 ^b^	13.62 ± 0.75 ^c^
Ferritin (ng/mL)	10.72 ± 4.10 ^a^	15.93 ± 8.66 ^b^	38.86 ± 15.34 ^c^
Haematocrit (%)	37.18 ± 2.43 ^a^	38.55 ± 2.48 ^b^	40.27 ± 2.60 ^c^
Mean corpuscular volume (fl.)	83.11 ± 5.97 ^a^	86.63 ± 4.30 ^b^	89.17 ± 3.79 ^c^
Red blood cell distribution width (%)	14.56 ± 1.36 ^a^	13.05 ± 0.78 ^b^	12.35 ± 0.50 ^c^
Transferrin (mg/dL)	338.69 ± 36.02 ^a^	352.15 ± 64.87 ^a^	277.81 ± 35.16 ^b^
Serum iron (μg/dL)	42.48 ± 13.06 ^a^	90.71 ± 39.40 ^b^	91.16 ± 33.03 ^b^
Transferrin saturation (%)	9.06 ± 2.98 ^a^	19.08 ± 9.12 ^b^	23.72 ± 8.84 ^c^
Soluble transferrin receptor (μg/mL)	2.27 ± 0.51 ^a^	1.49 ± 0.33 ^b^	1.16 ± 0.25 ^c^

Data are mean ± SD. Differences between the clusters were highly significant for all parameters (*p* ≤ 0.001, ANOVA). Different letters show differences between the clusters (*p* ≤ 0.01, Bonferroni *post hoc* test).

**Table 2.** Energy, total iron and food groups intakes according to the clusters.

Component	Cluster 1	Cluster 2	Cluster 3	ANOVA
Energy (Kcal/day)	2096 ± 474	2133 ± 594	2183 ± 645	NS
Proteins (% energy/day)	16.8 ± 3.3	16.1 ± 4.2	16.9 ± 9.5	NS
Carbohydrates(% energy/day)	40.4 ± 6.7	41.6 ± 7.2	40.3 ± 6.6	NS
Lipids (% energy/day)	39.2 ± 6.5	39.7 ± 7.1	40.9 ± 7.4	NS
Total iron (mg/day)	14.0 ± 5.7	13.2 ± 4.7	14.8 ± 5.7	NS
Cereals (g/day)	163.6 ± 65.0	171.2 ± 76.5	178.3 ± 72.7	NS
Legumes (g/day)	13.9 ± 31.4	7.8 ± 13.5	10.6 ± 18.2	NS
Vegetables (g/day)	235.2 ± 88.0	237.7 ± 118.7	221.7 ± 127.0	NS
Fruits (g/day)	207.9 ± 179.4	199.2 ± 190.9	186.3 ± 131.1	NS
Milk and dairy products (g/day)	435.1 ± 181.7	387.8 ± 168.5	372.0 ± 195.2	NS
Fish (g/day)	65.7 ± 44.7	42.2 ± 47.2	42.3 ± 47.6	NS
Eggs (g/day)	22.3 ± 14.8	24.7 ± 21.7	22.6 ± 19.0	NS
Red meat (g/day)	42.2 ± 48.4	35.5 ± 40.4	75.8 ± 66.0 *	<0.01
White meat (g/day)	23.6 ± 42.0	41.1 ± 49.5	38.5 ± 42.9	NS
Processed meat (g/day)	41.2 ± 53.0	36.2 ± 30.4	40.2 ± 31.0	NS

Data are mean ± SD; NS, non-significant;

* *p* < 0.05 compared to Cluster 2.

**Table 3.** Parameters of menstrual blood loss assessment and oral contraceptives use according to the clusters.

Parameter	Cluster 1	Cluster 2	Cluster 3	ANOVA
Age of menarche (years)	12.5 ± 1.3	12.8 ± 1.4	12.7 ± 1.6	NS
Menstrual cycle length (days)	27.9 ± 4.2	28.5 ± 3.7	30.0 ± 5.0	NS
Menstruation duration (days)	5.19 ± 1.06	4.92 ± 1.07	4.70 ± 0.99	NS
Days with intensive bleeding (days)	2.15 ± 0.55	2.03 ± 0.78	1.85 ± 0.63	NS
TTI (units)	5.52 ± 1.64	5.17 ± 1.59	5.14 ± 1.35	NS
MBLC (units)	279 ± 171	230 ± 117 *	206 ± 88 *	0.039
OC users (%)	16	28.1	54.5 *	0.001 #

TTI: Number of tampons and/or towels used in the day of more intensive bleeding; MBLC: Menstrual blood loss coefficient; OC: oral contraceptives; Data are mean±SD except for OC users, expressed in percentages; NS, non-significant;

# Pearson Chi-Square;

* *p* ≤ 0.05 compared to Cluster 1, *p* < 0.05 compared to Cluster 2.

**Table 4.** Genotype frequencies according to the clusters.

SNP	Genotype	Cluster 1	Cluster 2	Cluster 3
rs3811647	W(GG)	56.0%	51.7%	43.4%
	H(AG)	32.2%	43.1%	45.3%
	M(AA)	12.0%	5.2%	11.3%

rs1375515	W(AA)	56.0%	51.7%	45.3%
	H(AG)	36.1%	39.7%	47.2%
	M(GG)	8.0%	8.6%	7.5%

rs1799945 (H63D)	W(CC)	64.0%	58.6%	73.6%
	H(CG)	36.3%	37.9%	24.5%
	M(GG)	0.0%	3.4%	1.9%

rs1800562 (C282Y)	W(GG)	100.0%	91.4%	94.3%
	H(AG)	0.0%	8.6%	5.7%

rs1799852	W(CC)	68.1%	75.9%	81.1%
	H(CT)	32.0%	24.1%	18.9%

W: wild type homozygote; H: heterozygote; M: mutated homozygote; Pearson Chi-Square test among clusters was non-significant.

**Table 5.** Independent factors included in the categorical regression model that explained cluster belonging (*n* = 142, *R*^2^ = 0.142, *p* < 0.001).

Factor	B ± S.E *	Importance Coefficient (Rank)
Red meat (g/day)	0.301 ± 0.088	0.677 (1)
MBLC (units)	−0.149 ± 0.086	0.170 (2)
rs1800562 (C282Y)	0.150 ± 0.089	0.153 (3)

* Standardized slope ± standard error;

MBLC: Menstrual blood loss coefficient.
